# Affiliate Marketing in ophthalmological services

**DOI:** 10.22336/rjo.2022.5

**Published:** 2022

**Authors:** Consuela-Mădălina Gheorghe, Victor Lorin Purcărea, Iuliana-Raluca Gheorghe

**Affiliations:** * Department of Marketing and Medical Technology, “Carol Davila” University of Medicine and Pharmacy, Bucharest, Romania

**Keywords:** affiliate marketing, eyewear, ophthalmological products, consumer trust, affiliates

## Abstract

During the last decade, Internet has become an essential element in Marketing due to the benefits that come along with it, especially, in services that may promote and distribute products and services easily. In scientific literature, any activity that involved on online selling of a product or a service was classified as being e-commerce. In the last few years, since the pandemic started, many sellers had to find innovative distribution methods by implementing the principles of Affiliate Marketing (AM). Unfortunately, the implementation of AM principles in health care services has not gained too much attention because there are not so many specialists who would identify AM guidelines and take into consideration the full potential of this form of Marketing.

The aim of this review was to investigate the existing literature on health care services and Affiliate Marketing and to assess whether the principles of AM might be successfully applied in health care services, with a specific interest in Ophthalmology products.

Recent empirical proof of the efficient contemporary Marketing approach revealed that the key of economic growth is the sharing of knowledge, expertise, skills, collaboration and networking, namely, a partnership that would bring mutual benefits for all the parties involved, as well as the creation of added value for consumers [**1**]. These partnerships will enable know-how and cooperation on a long period of time, which, in its turn, will improve quality to what the partnership offers, new product development in terms of innovation and will apply a suitable risk management [**2**]. In the pandemic context, Affiliate Marketing (AM) has been the effective modern form of partnering on the Internet for Marketing purposes [**1**].

As much profitable as it may prove to be, few empirical research studies have been conducted on topics related to AM. As the scientific literature revealed, only 33% of the specialists in Marketing, who were involved in e-commerce, were poorly informed about the AM concept [**3**] because there were unexpected negative consequences associated with what was considered good practices in Marketing, such as inconsistent branding through many affiliates, which has eventually led to consumer distrust and negative reputation about an organization among them [**4**]. However, in some industries, especially in tourism, e-commerce has evolved to AM, at the same time, contributing significantly to the organizations’ online Marketing strategies [**5**]. Thus, in the pandemic context, many organizations had to change their perspectives and, in fact, had to reinvent in order to survive on the market. 

The aim of this review was to discuss if the principles of AM might be successfully assessed in other industries, apart from tourism, as for instance, in health care services. To our knowledge, there is limited literature on the concept of AM and there are very few studies that focused on linking the AM concept to the health care services. 

## The concept of Affiliate Marketing (AM)

In the context of high Internet selling products, namely, e-commerce, a new type of Marketing, Affiliate Marketing (AM), emerged, which is the fastest growing method to increase sales [**6**]. AM has become an important source of revenue especially during the pandemic, in fact being a key source of online income for many organizations and individuals. Health care services are classified as being pure, however, there are some medical specialties that transform the provided services into a complementary mixture of services and products, as for instance, the Ophthalmology services [**7**].

AM is defined as the online act of promoting someone else’s products and services to earn commissions from the sales provided [**8**]. Thus, the broadest description of an AM activity relates to the following: an affiliate, such as an advertiser or third-party distributor, signs an agreement with a company, namely an organization, to promote the seller’s products on the affiliate’s website in return for a commission provided by the seller, but this traditional form of AM has suffered several changes depending on the industry. In the health care context, and, specifically, in Ophthalmology, an organization agrees with several third parties to sell contact lens or eye-glasses online or offline (in store). 

The primary objectives of AM focus on promoting and selling products and services through a distribution online shop, raise the web-based traffic of the seller as well as generate purchase transactions from online users in return for a commission that will be received by the affiliate [**9**]. The payment conditions for the affiliate depend on the industry and the principles of AM. There are several types of commission payment, as follows [**10**,**11**]: 

The pay-per-sale relies on the fact that the commission will be paid depending on the number of purchases generated by the affiliates;

- The pay-per-lead relies on the fact that the commission will be paid to the affiliates depending on the number of new consumers acquired;

- The pay-per-click is reflected in a pre-determined flat fee, regardless of the number of generated sales;

- The cost-per-thousand refers to the commission that is paid to affiliates for every 1000 online user visits. 

In this sense, AM is a source of income an affiliate may gain, while working remotely [**12**]. Even if there are several opinions about AM not being a truly comprehensible Marketing strategy, in comparison to other strategies (i.e., email Marketing) [**13**], it is expected to generate high revenues in the coming years, especially in the pandemic context. 

## The benefits and pitfalls of AM

**Fig. 1:**
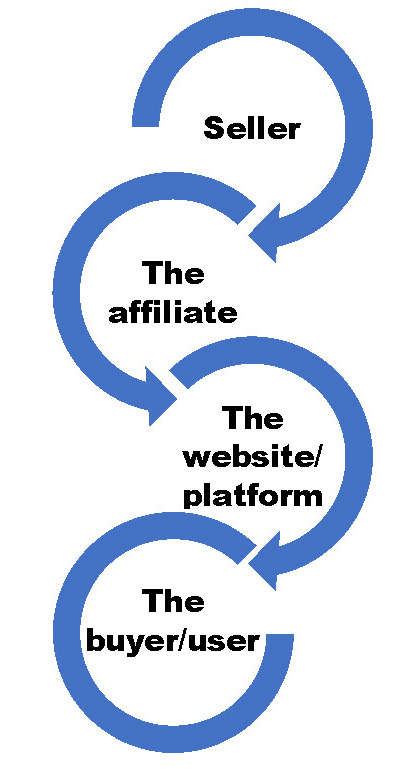
Parties involved in an AM activity

The process of AM generally involves four parties, namely the seller, the platform, the affiliate and the user or the buyer (**Fig. 1**).

The seller may be an entity, such as an organization or an individual, that provides a product or a service online or offline, and the affiliate is a person or a third party with the role of generating content or promoting a service or a product through different online or offline methods.

In an online environment, the connection between the seller and the affiliate is made through a platform or the website of the affiliate. The platform includes products and services, which require promotion through special links, provided by the affiliate in order to direct a potential user or buyer to the desired product or service on the seller’s website or the affiliate’s website. The buyer is the end user of the product or service, and he/ she is not aware of the AM activity between the seller, the platform, and the affiliate.

Depending on the parties involved in the AM activities, AM offers several benefits that would also reveal the motivation of implementing AM in practice. For sellers, AM contributes significantly to the decrease of the expenditures regarding advertising and it attracts potential new consumers with low costs [**14**]. In addition, AM is also beneficial for new websites or start-ups that do not have the necessary financial resources to spend on advertising their products or services. For the affiliate, AM offers an opportunity to make money by using his/ her personal networks or professional networks, online networks, and Social Media networks. More exactly, affiliates are highly relevant and become efficient because they reach specific target segments [**15**]. For the potential consumers, AM provides an exposure to new products or services from trustworthy sources [**16**]. In fact, AM becomes a valuable Marketing strategy if the seller and the affiliate investigate the consumers’ perceptions about it, in terms of trusting the online information sources [**17**]. 

We highly support gaining a deeper understanding of the consumers’ acceptance of AM activities and investigating the methods affiliates would use to attract potential consumers in order to build trust and, ultimately, to generate profit [**8**]. 

All in all, AM may prove to be a sustainable activity, leading to considerably lower wastages in what concerns the cost/ performance ratio [**8**].

Nevertheless, there are some pitfalls that need to be addressed when implementing AM strategies. The most important risks are the seller’s because he/ she cannot monitor and control the affiliates’ activities thoroughly and these may end with negative effects in the shape of affiliate’s deceptive behavior [**18**] and specific related brand issues such as reputation and image [**19**]. Thus, being the first touch point with the potential consumers, affiliates have to offer significant online information to positively impact their image as well as the seller’s image [**17**]. 

From an affiliate’s perspective, the main risk in applying AM strategies lies in not gaining sufficient financial resources to cover the AM running expenses, as for instance, providing a sophisticated and appealing website or investing constantly in SEO (search-engine-optimization) strategies. Hence, affiliates need to deeply understand how consumers select their website, and what are the elements that would build consumer trust at an operational level, and would apply AM efficiently and profitably. 

From a consumer’s perspective, the risks of AM activities relate to trust. The elements that can build trust for consumers on an affiliate’s website with specific ophthalmologic products are further discussed. 

## The principles of AM in Ophthalmological services

The interest in adopting AM practices in health care services is still in its infancy because AM has not raised any interest in users or buyers. The motivation steams from the poor reputation of AM, especially related to the intrusive mass advertising and even commission fraud [**20**]. However, as far as ophthalmological organizations are concerned, third parties and affiliates that can benefit from AM are the ones selling optical products, especially eyeglasses, sunglasses, lenses, contact lenses, etc. For these kinds of products, it is important to have a website where they can be first advertised and afterwards bought by potential consumers. Hence, the two product categories dedicated to Ophthalmology are connected to contact lenses and eyewear (glasses). 

**• Contact lenses**

According to Fogel and Zidile [**21**], people are increasingly purchasing contact lenses over the Internet. Their study revealed that people who purchased contact lenses over the Internet less often adhered to the FDA recommendations in comparison to the persons who purchased them from a physician’s private office. 

**• Eyewear (eye glasses**)

According to the 2020 Internet Influence Report from The Vision Council [**22**] in the U.S.A., the number of adults who purchased eyeglasses over the Internet increased from 22% to 44%. This situation appeared even before the COVID-19 pandemic and it continued to increase during the pandemic. The same study revealed that people usually use two different types of websites when they decide to purchase eyeglasses over the Internet, in fact, depending on many trustful elements that relate to the design of the website and the credibility of the affiliates.

Trust has been identified to be a key factor in e-commerce, namely when establishing a relationship between a consumer and a commercial website [**23**], and, at the same time, has been widely researched in terms of antecedents and outcomes [**24**]. However, consumer trust in the context of AM has received limited attention, especially in the health care field. As such, the trust concept has been a core element in building online successful consumer-seller interactions, and it has significantly contributed to influencing consumers’ online behavior [**25**], as well as in raising consumer loyalty [**26**]. In the context of health care services, affiliates should be interested in building trustful relationships with their buyers because they reduce the consumer perceived risks and increase the reliability of perceived information [**27**], at the same time raising the spread of word-of-mouth [**26**]. Spreading WOM in an offline context may be the spark for spreading eWOM [**28**] in an online context and build not only trust in the affiliate, but also in the seller, and, finally, generate profit and build a positive online reputation [**29**]. In order to achieve these objectives, research on the antecedents of trust need to be addressed in an e-commerce context, but rather in an AM context. 

We argue that the antecedents of a consumer’s trust in an affiliate in the health care context, and, implicitly, in an ophthalmologic setting, should be investigated depending on several criteria that relate to the credibility of the affiliate and to the design characteristics of a website. 

• Credibility antecedents of the affiliate

**Fig. 2:**
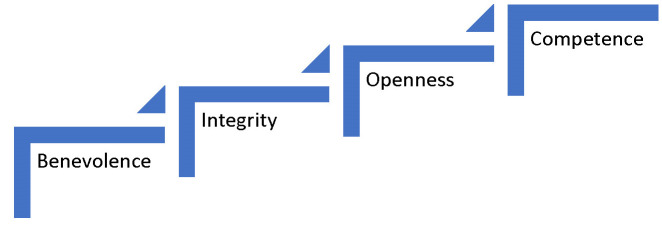
Credibility characteristics of an affiliate

Several studies that focused on online trust research have revealed that the most frequently trusting consumer belief refers to benevolence, integrity, openness, and competence (**Fig. 2**) [**30**].

In the AM context, benevolence refers to the affiliates’ handling personal sensitive information provided by the buyer in a legal and non-fraudulent manner [**30**,**31**]. The sensitive information consists of credit card and other personal payment details, but most optical websites offer information about the products and redirect the buyer to the seller’s website, so affiliates will not handle sensitive information most of the times. However, some structural assurances may also raise the trust of the affiliates in the buyers if they request credit card guarantees or verification seals. 

Competence encompasses elements that concentrate on the affiliate’s ability to fulfill the promise, as communicated initially to the buyer, through the website or the platform [**32**]. In the AM context, competence reflects the buyer’s confidence that the affiliate will provide and deliver the adequate outcome, in our case, sunglasses or contact lenses, specifically to fulfill an expectation that is a very personalized desire blended with the skills of the affiliate, the optometrist. 

Integrity and openness should be approached together in the AM context. In an online setting, integrity refers to the reliability, honesty, and ethical traits of the seller, whereas the openness is defined as presenting all the information without deliberately withholding anything that may raise suspicion [**23**]. In the AM context, integrity could refer to the truthfulness and honesty of the information provided, as for example, in our case, the optometrist offers enough information about where the eyeglasses were produced and other technical information. Openness may refer to the information sharing process on the platform or the website, as for instance, the optometrist should openly disclose the prices of the contact lenses without asking the buyer to send a private message. 

• Credibility antecedents of the website/ platform

The e-commerce literature has acknowledged the crucial role a website interface design has on building consumer trust [**26**]. Thus, the comparison to a salesperson is not far from the truth if a website has an enhancing trustworthy interface [**32**]. As in the context of e-commerce, an AM design interface will induce trust in consumers at various levels: graphical layout, the structure design, and the content design [**33**].

According to AM, the web design interface must be easily used by consumers and great importance should be attached to the graphical representations in terms of pictures and color schemes, which will help in visualizing the product. Therefore, when selling an optical product, the color schemes should be simple and clear, inspiring calmness and trust. 

The layout of the website or the platform should have clear and easily comprehensible steps for site navigation, such as a simple structure homepage. Some structural design elements may trigger the consumer’s trust if the affiliates constantly and explicitly inform consumers about the usage of personal data by proactively communicating statements about privacy and confidentiality as well as visibly display credit card information requirements. Moreover, affiliates should also provide contact details in order to give consumers an opportunity to address questions in case issues occur when they use the platform/ website. 

It is also worth mentioning that other two antecedents may raise the trust in the affiliate, as follows:

• Website/ platform reputation is a trust determining antecedent for consumers in specific cases of product failure and guarantees. Several consumers buy eyeglasses and contact lenses from the first website/ platform that pops-up whenever they search online for these products, using a search engine. In other words, from a consumer’s perspective, if the website/ platform is ranked among the top three to five results returned by a search engine, it represents an indicator of trust. 

• The partnership agreement between the affiliates and the seller should ensure shared understanding and affinity towards the same target markets. 

## Conclusion

AM has been defined in the scientific literature as being close to the meaning of e-commerce, but, given the particularities of each industry, it emerged as a new independent field, especially during the pandemic, when many sellers had to reinvent their distribution strategies. This was the case of health care services that did not fit entirely in the AM typical activity. However, in this review, we have argued that AM could be successfully applied in some medical specialties, such as Ophthalmology. The key elements in providing efficient AM activities should focus on building consumer trust and reliable partnerships between affiliates and sellers. 

**Conflict of Interest statement**

The authors state no conflict of interest.

**Acknowledgements**

None.

**Sources of Funding**

None.

**Disclosures**

None.
